# Leveraging Network Target Theory for Efficient Prediction of Drug‐Disease Interactions: A Transfer Learning Approach

**DOI:** 10.1002/advs.202409130

**Published:** 2025-01-28

**Authors:** Qingyuan Liu, Zizhen Chen, Boyang Wang, Boyu Pan, Zhuoyu Zhang, Miaomiao Shen, Weibo Zhao, Tingyu Zhang, Shao Li, Liren Liu

**Affiliations:** ^1^ Department of Molecular Pharmacology, National Clinical Research Center for Cancer, Key Laboratory of Cancer Prevention and Therapy, Tianjin, Tianjin's Clinical Research Center for Cancer Tianjin Medical University Cancer Institute & Hospital Tianjin 300060 China; ^2^ Institute for TCM‐X, Department of Automation Tsinghua University Beijing 100084 China; ^3^ Henan Academy of Sciences Henan 450046 China

**Keywords:** cancer, drug combination, drug‐disease interaction (DDIs), few‐shot learning, network target

## Abstract

Efficient virtual screening methods can expedite drug discovery and facilitate the development of innovative therapeutics. This study presents a novel transfer learning model based on network target theory, integrating deep learning techniques with diverse biological molecular networks to predict drug‐disease interactions. By incorporating network techniques that leverage vast existing knowledge, the approach enables the extraction of more precise and informative drug features, resulting in the identification of 88,161 drug‐disease interactions involving 7,940 drugs and 2,986 diseases. Furthermore, this model effectively addresses the challenge of balancing large‐scale positive and negative samples, leading to improved performance across various evaluation metrics such as an Area under curve (AUC) of 0.9298 and an F1 score of 0.6316. Moreover, the algorithm accurately predicts drug combinations and achieves an F1 score of 0.7746 after fine‐tuning. Additionally, it identifies two previously unexplored synergistic drug combinations for distinct cancer types in disease‐specific biological network environments. These findings are further validated through in vitro cytotoxicity assays, demonstrating the potential of the model to enhance drug development and identify effective treatment regimens for specific diseases.

## Graphical abstract

This study introduces an innovative transfer learning model that revolutionizes drug discovery by integrating network target theory with deep learning. The model successfully identifies over 88,000 drug‐disease interactions and predicts effective drug combinations with high accuracy. Through disease‐specific biological networks and experimental validation, it demonstrates remarkable potential for accelerating drug development and optimizing treatment strategies, particularly in cancer therapeutics.

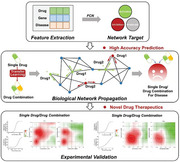

## 1 Introduction

Drug development remains a time‐consuming and expensive process, with estimated costs exceeding $2.6 billion per successful drug.^[^ ^1^, ^2^ ^]^ Computational methods have emerged as promising solutions to expedite early‐stage development and reduce these substantial costs. Among these approaches, drug repurposing has gained particular attention for its cost‐effectiveness and reduced development timeline^[^ ^3^, ^4^, ^5^ ^]^ However, the complexity of human diseases often requires therapeutic strategies beyond single‐drug interventions, as demonstrated by the superior efficacy of combination therapies in conditions like cancer.^[^ ^6^, ^7^, ^8^, ^9^, ^10^ ^]^ While high‐throughput screening can evaluate some drug combinations, its application is limited by cost, time, and the lack of suitable human cell line models for many diseases.^[^ ^11^, ^12^ ^]^ These challenges highlight the crucial need for computational algorithms that can effectively predict both single and combination drug‐disease interactions.

Current virtual studies investigating drug‐disease interactions (DDIs) employ various computational approaches, with deep learning emerging as a powerful paradigm alongside traditional methods like matrix factorization and network propagation. Matrix factorization techniques, while useful for decomposing large sparse matrices into manageable components, face challenges with computational intensity and accuracy due to missing values.^[^ ^13^, ^14^, ^15^, ^16^ ^]^ Recent deep learning approaches have demonstrated significant advances in DDI prediction: graph neural networks (GNNs) have shown promise in capturing complex molecular interaction patterns,^[^ ^17^, ^18^ ^]^ while transformer‐based architectures have effectively learned drug‐disease representations from heterogeneous biological data.^[^ ^19^ ^]^ Multi‐modal deep learning frameworks have successfully integrated diverse data types, including molecular structures, gene expression profiles, and protein‐protein interaction networks.^[^ ^19^, ^20^ ^]^ However, challenges persist regarding data quality, model interpretability, and the need for large training datasets.^[^ ^21^, ^22^, ^23^ ^]^ Network propagation methods have shown promise in disseminating information within biological networks to identify relationships among proteins, genes, drugs, and diseases, yet issues related to data accuracy and verification remain unresolved.^[^ ^24^, ^25^, ^26^, ^27^, ^28^ ^]^

Network pharmacology represents a paradigm shift in understanding drug‐disease relationships, offering a comprehensive, systems‐based approach that combines computational biology, pharmacology, and systems biology.^[^ ^29^, ^30^, ^31^, ^32^ ^]^ Unlike traditional pharmacology's single‐target focus, network pharmacology views diseases as the result of complex molecular interactions, where multiple targets are involved. This holistic perspective enables researchers to explore drug‐disease relationships at a network level, providing insights into both individual drug‐target interactions and how drugs may act on multiple targets within biological systems to modulate disease progression. Central to this approach is the theory of network targets, which was first proposed Li et al. (2011) to address the limitations of traditional single‐target drug discovery approaches.^[^ ^33^ ^]^ Network targets represent a novel concept that views the disease‐associated biological network as a therapeutic target, rather than focusing on individual molecules. This theory posits that diseases emerge from perturbations in complex biological networks, and effective therapeutic interventions should target the disease network as a whole. Network targets can include various molecular entities such as proteins, genes, or pathways that are functionally associated with disease mechanisms, and their interactions form a dynamic network that determines disease progression and therapeutic responses.

Recent advances in artificial intelligence and big data analysis have enabled comprehensive exploration of multi‐level biological networks aligned with network target theory, leading to several innovative methodological breakthroughs. For instance, NIMS has demonstrated remarkable effectiveness in identifying synergistic drug combinations by leveraging disease‐specific biological networks as therapeutic targets and translating drug relationships into network‐level interactions.^[^ ^34^ ^]^ Similarly, FordNet has showcased the power of integrating both phenotype (clinical symptoms) and molecular information to predict Traditional Chinese Medicine formula efficacy, embracing a holistic perspective to understand complex interactions between herbs, compounds, targets, and diseases.^[^ ^35^ ^]^ The field has further evolved with the development of sophisticated approaches like drugCIPHER,^[^ ^36^ ^]^ which integrates pharmacological and genomic information to predict drug‐target interactions on a genome‐wide scale. By incorporating drug therapeutic similarity, chemical similarity, and protein‐protein interaction networks, such methods generate comprehensive biological fingerprints for drugs, enabling more accurate prediction of potential drug targets. Recent research has also demonstrated the value of network target theory in understanding complex disease mechanisms, as exemplified by studies identifying functional synergistic modules in inflammation‐induced tumorigenesis through the integration of gene coexpression networks and CRISPR‐Cas9‐based genetic interaction analysis.^[^ ^37^ ^]^

Despite these advances, several challenges persist in current drug‐disease prediction methods. A significant obstacle lies in the pronounced imbalance between known and unknown associations, highlighting the importance of appropriate negative sample selection. Furthermore, integrating biological networks with additional prior knowledge presents both an opportunity and challenge for improving prediction accuracy. The application of knowledge gained from large individual datasets to predict drug combinations in smaller datasets also poses significant methodological challenges.

In this study, we present a novel methodology based on network targets that integrates various biological molecular networks with transfer learning techniques to forecast DDIs. Our approach demonstrates enhanced predictive accuracy for individual DDIs compared to similar algorithms. Moreover, our algorithm enables direct prediction of drug combinations and generate more precise results within disease‐specific scenarios.

## 2 Experimental Section

### 2.1 Datasets Construction

#### 2.1.1 Drug‐Target Interaction Dataset

The comprehensive dataset was obtained from DrugBank,^[^ ^38^ ^]^ and entries related to drug‐target interactions were carefully selected. The structural representation of each pharmaceutical agent, denoted by the SMILES^[^ ^39^ ^]^ notation, was retrieved from the PubChem repository. In total, an extensive corpus of 16508 drug‐target interaction entries was extracted from the dataset. Following the established classification schema of drug‐target interaction patterns,^[^ ^40^ ^]^ these entries were systematically divided into three distinct categories: 2024 were classified as activation interactions, 6969 as inhibitory interactions, and the remaining 7525 entries were identified as non‐associative interactions. All three categories of interactions (activation, inhibition, and irrelevant) were collected and retained throughout our model construction. This decision was based on two key considerations: First, in real biological systems, drugs can exhibit multiple modes of action on their targets, and excluding any interaction type would limit our model's ability to capture these complex relationships. Second, during the network target propagation phase, both activation and inhibition effects play crucial roles in how drug perturbations propagate through molecular networks.

#### 2.1.2 Disease Dataset and Disease Embedding Model

MeSH^[^ ^41^ ^]^ descriptors, curated by the United States National Library of Medicine, were employed to meticulously extract disease‐related information. These descriptors are systematically organized into a hierarchical lexicon that encompasses a wide range of concepts, spanning from chemical compounds to pathological conditions. In a previous study^[^ ^42^ ^]^ the intricate MeSH taxonomy was effectively transformed into an interconnected topical network using advanced graph embedding techniques, thereby incorporating a network that delineates interrelations between diseases. Consequently, this comprehensive network with 29349 nodes and 39784 edges served as the foundational dataset for the subsequent extraction and analysis of disease‐related information.

#### 2.1.3 Drug‐Disease Interaction Dataset

The dataset detailing compound‐disease interactions was obtained from the Comparative Toxicogenomics Database.^[^ ^43^ ^]^ The delineation of diseases within this collection is derived from both MeSH and OMIM databases. A rigorous filtration process was applied to this dataset based on three criteria: 1) the existence of direct empirical evidence supporting the compound‐disease interaction; 2) the inclusion of the disease in the MeSH taxonomy; 3) the availability of the drug's SMILES notation on the PubChem platform. Following this stringent selection process, a refined dataset was created, comprising 88161 interactions between drugs and diseases, involving 7940 pharmacological agents and 2986 distinct disease conditions.

#### 2.1.4 Combination Drug Dataset

An assemblage of combination drug therapies was compiled from authoritative sources, namely DrugCombDB,^[^ ^44^ ^]^ Therapeutic Target Database (TTD),^[^ ^45^ ^]^ and the National Comprehensive Cancer Network (NCCN). Following a meticulous reconciliation process aligning the therapies with standardized nomenclature for drugs and diseases, a total of 301 combination drug therapies were identified. From this collection, a subset of 104 therapies was carefully selected based on the criterion that each constituent drug‐disease pair was included in the previously curated drug‐disease interaction dataset.

#### 2.1.5 PPI Network

The Protein‐Protein Interaction (PPI) network, essential for the gene embedding procedure, was derived from the STRING.^[^ ^46^ ^]^ database, a comprehensive repository of known and predicted protein interactions. This extensive network incorporates 19622 genes and a vast array of protein interaction relationships numbering at 13.71 million. For the drug's random walk analysis, we utilized the Human Signaling Network (Version 7)^[^ ^47^ ^]^ as the signed PPI network. It is meticulously annotated with 33398 activation interactions and 7960 inhibition interactions involving a total of 6009 genes. This signed network provides a detailed map of signaling pathways, that facilitates nuanced insights into the molecular interplay influenced by pharmaceutical agents.

#### 2.1.6 Cancer Specific Data

The disease compendium from The Cancer Genome Atlas (TCGA) was retrieved from the UCSC Xena database,^[^ ^48^ ^]^ which includes a diverse spectrum of cancer expression profiles alongside GTEX normal tissue expression data, served as a valuable dataset for constructing cancer‐specific networks. The selection of cancer types for inclusion was based on two crucial criteria: (1) the concurrent availability of expression data for both cancerous and normal tissue samples; (2) representation within the drug‐disease interaction dataset, with a minimum requirement of 20 therapeutic agents. Adhering to these stringent prerequisites, we meticulously chose 18 cancer types for in‐depth network construction and subsequent analysis.

### 2.2 Feature Extraction for Drug, Disease, and Gene

#### 2.2.1 Drug Embedding

The InfoGraph^[^ ^49^ ^]^ algorithm was employed in this study to generate low‐dimensional embeddings of pharmaceutical compounds. InfoGraph operates within an unsupervised learning framework, aiming to maximize mutual information between the graph representation and its substructures, effectively capturing the topology of the graph. Implemented using Torchdrug,^[^ ^50^ ^]^ we utilized the Graph Isomorphism Network (GIN)^[^ ^51^ ^]^ model as the encoder. For training, we utilized the ZINC2 m^[^ ^52^ ^]^ dataset which provided the molecular structures. After converting drugs' SMILES notations into molecular structure graphs via RDKit, these graphs were then fed into InfoGraph, resulting in 300‐D vector representations for each molecule, thereby condensing complex structural data for computational analysis.

Specifically, given a compound's SMILES representation S, the molecular M is obtained by

(1) $$M=Mol\;From\;Smiles\left(S\right)$$

Then each compound is embedded by a 300‐D representation

(2) $$Drug\;Embedding=Info\;Graph\left(M\right)$$

#### 2.2.2 Disease Embedding

The node2vec^[^ ^53^ ^]^ method was employed for the low‐dimensional embedding of the MeSH network. By leveraging biased random walks, node2vec generates a multitude of walk sequences, treating walks as sentences and nodes as words in an analogous manner. Subsequently, the word2vec^[^ ^54^ ^]^ algorithm is applied to derive compact vector representations of these nodes. The MeSH network, labeled as *G_M_* =  (*V_M_*, *E_m_*), consists of 29349 subject terms *V_m_* interconnected by 39784 edges *E_m_*. This embedding process was executed using the PyTorch Geometric^[^ ^55^ ^]^ package, tailored for analyzing graph‐structured data. Consequently, each MeSH term was converted into a 300‐D vector, capturing both semantic and relational aspects in a computationally efficient manner.

(3) $$G_{M}^{\prime }=\left(V_{M}^{\prime },E_{m}\right)=node2vec\left(G_{M}\right)$$

(4) $$\mathrm{Value}\left(v\right)\in R^{300},\mathrm{for}\;\mathrm{each}\;v\in {V^{\prime }}_{M}$$

During the training process, a consistent embedding dimension of 300 was set to provide a comprehensive and efficient representation of each node. To capture diverse network aspects, each node underwent 20 samplings. Thorough network exploration, was achieved by employing a walk length of 40 steps, which primarily focused on immediate contextual links. By setting the return and exploration parameters at 1, an optimal balance between revisiting nodes and discovering new paths was attained, thus achieving a holistic view of the network's dynamics.

#### 2.2.3 Gene Embedding

The node2vec method was consistently applied during the embedding process of the PPI network from the STRING database, following the same approach used for the MeSH network. To ensure uniformity in the embedding process, consistent training parameters were employed for both networks. Consequently, each gene in the PPI network was transformed into a 300‐D vector using identical embedding dimension, node sampling frequency, walk length, and return and exploration parameters as established for the MeSH network. This approach facilitated a harmonized and efficient representation of both networks for subsequent analyses.

### 2.3 DNN‐Based Drug‐Target Prediction

In this study, we employed a fully connected neural network implemented using PyTorch to predict drug‐target interactions. The input of the network is constructed by concatenating vectors that represent unique drug‐gene pairs. After rigorous hyperparameter tuning using grid search, we identified the optimal model configuration. The number of layers was selected from 2, 3, and 4, with a 3‐layer architecture yielding the best performance. The number of neurons in each layer was chosen from various configurations, settling on 600, 1024, and 3 neurons respectively. The Adam optimizer was chosen over SGD with momentum and RMSprop because it demonstrated superior performance in this specific context. The learning rate and weight decay were fine‐tuned between the values of 0.01 and 0.0001 and the optimal configuration was found to be a learning rate of 0.0001 and a weight decay of 0.001, which yielded the best results. The Exponential Linear Unit (ELU)^[^ ^56^ ^]^ was selected as the activation function due to its effectiveness in facilitating non‐linear transformations while avoiding gradient vanishing problem. To enhance the model generalization ability and prevent overfitting, batch normalization and a dropout rate of 20%, which was chosen from trials from 5% to 50%, were integrated into the model structure.

One of the notable challenges in this study was the presence of an imbalance within the training dataset, characterized by a disproportionately high number of inhibitory and non‐relevant interactions compared to activation interactions. To effectively counteract this issue, we incorporated FocalLoss^[^ ^57^ ^]^ as our chosen loss function. Focal Loss effectively addresses this imbalance through its modulating factor (1 − *p_t_*)^γ^, where *p_t_* is the probability of the correct class. This factor automatically down‐weights the loss contribution from easy examples (common interactions that the model predicts correctly with high confidence) while maintaining higher loss values for difficult examples (rare interactions that the model struggles to predict correctly). Specifically in our dataset, where activation interactions are the minority class, Focal Loss ensures these rare but important cases receive adequate attention during training. When the model becomes very confident in predicting the majority classes (inhibitory and non‐relevant interactions), the modulating factor reduces their impact on the overall loss, preventing them from dominating the learning process. This allows the model to maintain focus on improving its prediction accuracy for the underrepresented activation interactions. Focal Loss was chosen over alternative approaches like resampling or class weighting because it dynamically adjusts the learning focus based on prediction difficulty, without requiring artificial manipulation of the dataset structure or batch composition, which could introduce bias or computational overhead. Therefore, FocalLoss is particularly adept at handling imbalances in datasets by prioritizing training instances that are difficult to classify, thereby equalizing the influence of different classes despite their uneven representation. This approach proved crucial in mitigating the imbalance and enhancing the model's performance across all categories of interactions.

During the training phase, the Adam optimizer was employed due to its efficiency in dealing with large datasets and complex parameter spaces. To ensure a steady convergence, the learning rate was set at 0.001, while a weight decay of 0.0001 was utilized to address overfitting issues. In order to achieve precise prediction results, distinct thresholds were applied: an inhibitory interaction threshold of 0.88 and an activation interaction threshold of 0.44. By classifying interactions based on these thresholds, the model's accuracy was enhanced and imbalances in the dataset were effectively handled.

### 2.4 Biological Network Diffusion Simulation for Single Drug and Drug Combinations Based on Random Walk

The random walk method was utilized to simulate the effects of single and combination drug therapies. For a single drug with multiple targets, a multi‐start‐point random walk was conducted, wherein each target served as an individual start point. This approach effectively captures the drug's impact on its various targets within the network. In the case of drug combinations, separate multi‐start‐point random walks were conducted for each drug in the same signed PPI network, without resetting initial values between drugs. This method takes into account the synergistic effects of combining drugs and accurately reflects the intricate dynamics that arise from interactions among multiple drugs within a shared network.

In this study, the random walk strategy incorporates two key coefficients: a restart coefficient (α) set at 0.3, which allows for the possibility of returning to the starting node at each step, and a decay coefficient (β) set at 0.95, controlling the rate at which influence diminishes over successive steps. The direction of change in node values is determined by both the drug's effect (activating or inhibiting) on starting nodes and the properties of edges in the signed PPI network. The walk is configured with 15 epochs and a length of 15 steps per start node, enabling thorough network exploration and capturing the subtle effects resulting from the drug's action.

Initially, all nodes in the signed PPI network are set to zero.

(5) $$G_{P}=\left(V_{P},E_{P}\right)$$

(6)

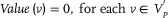




For start targetT,defineSing(T)

$\;=\left\{\begin{array}{c} 1,\text{if the drug's effect of}\;T\;\text{is activation}\\ -1,\;\text{if the drug's effect of}\;T\;\text{is inhibition} \end{array}\right..$

When the walk progresses to the *n^th^* step and arrive at node *v_i_*, the path walked is

(7) $$path=(e_{1},e_{2},\text{…},e_{n})$$

Define $p\;\left(e\right)=\left\{\begin{array}{c} 0,if\;the\;interaction\;of\;edge\;e\;is\;activation\\ 1,if\;the\;interaction\;of\;edge\;e\;is\;inhibition \end{array}\right.$, then

(8) $$Value\left(v_{i}^{n}\right)=Value\left(v_{i}^{n-1}\right)+\beta^{n}\times Sign\left(T\right)\times {\left(-1\right)}^{\Sigma_{i=1}^{n}p\left(e_{i}\right)}$$

The outcomes of the random walk are directed into a gene set comprising 18977 genes. Given that the signed PPI network includes a few nodes outside this gene set, which may potentially be connected to the target gene set, we did not initially filter the network to only retain the nodes within the gene set. This approach allows for a comprehensive exploration of the network. After completing the walk, the results are aligned with the 18977‐gene set, thereby enhancing both accuracy and relevance.

### 2.5 Drug‐Disease Interactions Prediction

#### 2.5.1 Dataset Division

The drug‐disease interaction prediction model utilizes an input vector, consisting of the concatenation of the random walk result vector and the disease vector, with a length of 19277. This comprehensive model encompasses 7940 drugs and 2986 diseases, resulting in over 23 million potential combinations of drug‐disease interactions (**Table** **1**). However, only 88161 interactions are currently known while the vast majority remains unknown. Existing models employ different methods to handle these unknown samples: LAGCN^[^ ^18^ ^]^ and SCMFDD^[^ ^58^ ^]^ considers all unknown samples as negatives; CTST,^[^ ^59^ ^]^ GFPred^[^ ^60^ ^]^ and CBPred^[^ ^61^ ^]^ randomly selects a number of negative samples equal to the number of positive samples; LRSSL,^[^ ^62^ ^]^ MBiRW^[^ ^63^ ^]^ and HGBI^[^ ^64^ ^]^ primarily utilize positive samples for training. Each approach offers a unique strategy to address the imbalance between known and unknown interactions in the dataset.

**Table 1:** The number of drugs, diseases, and their pairs.

Drugs	Diseases	Drug‐Disease Interactions	Drug‐Disease Pairs
7940	2986	88161	23708840

In our proposed method, we initially considered classifying all unknown samples as negative. However, this led to an extremely imbalanced positive‐to‐negative sample ratio of 1:268. Such imbalance could cause computational strain, poor predictive performance, and a lack of sufficient testing samples. To address this, we employed the Synthetic Minority Over‐sampling Technique (SMOTE)^[^ ^65^ ^]^ to artificially increase the number of positive samples to balance the dataset. The process began by selecting a subset of negative samples from the pool of over 23 million drug‐disease interactions, which was ten times the number of positive samples. This subset was then merged with the existing positive samples to create a comprehensive dataset. From this dataset, 80% was randomly chosen as the training set while the remaining 20% was allocated for validation (**Table** **2**). The SMOTE method was specifically applied to the training set in order to augment the count of positive samples and rectify the initial imbalance.

**Table 2:** The amount of positive and negative samples in different datasets.

	Training Set (original)	Training Set (SMOTE)	Validation Set
Positive samples	70553	705264	17595
Negative samples	705264	705264	176359

#### 2.5.2 Model Construction

By employing a grid search approach for hyperparameter tuning similar in drug‐target prediction section, we developed a 6‐layer fully connected neural network (FCNN) using PyTorch to predict drug‐disease interactions. The network architecture included hidden layers with 4096, 1024, 256, and 64 neurons correspondingly, while the input and output layers contained 19277 and 1 neuron respectively. The number of neurons in the input layer represents the combined feature numbers of drugs and diseases, whereas the output layer provides a drug‐disease match score indicating effectiveness (score > 0) or no relation (score < 0). Leaky ReLU^[^ ^66^ ^]^ was employed as an activation function in all hidden layers due to its effectiveness in mitigating the “dying ReLU” problem by allowing a small gradient for negative inputs. To address overfitting concerns, batch normalization, and a 50% dropout rate were implemented. Model training utilized binary cross‐entropy loss function (BCEWithLogitsLoss) and was optimized using the Adam optimizer with both learning rate and weight decay set at 0.001.

### 2.6 Drug‐Disease Interaction Prediction‐Based on Disease‐Specific Networks

#### 2.6.1 Cancer‐Specific Network Construction

The present study aimed to investigate the variability of Protein‐Protein Interaction (PPI) networks across different human tissues and disease conditions.^[^ ^67^ ^]^ In contrast to previous approaches using identical PPI networks, this study investigated the impact of employing disease‐specific PPI networks on the accuracy of drug‐disease interaction predictions. To achieve this objective, the Cancer Genome Atlas (TCGA) dataset was employed. Differential expression genes were identified by analyzing cancer sample data and corresponding normal tissue data for each specific cancer type. Subsequently, the Weighted Gene Co‐expression Network Analysis (WGCNA)^[^ ^68^ ^]^ tool was used to calculate the gene correlation matrix. Gene pairs with a *P*‐value less than 0.05 were selected as network edges, and positive or negative correlation indicated promotive or inhibitory interactions, respectively. Two TCGA disease‐specific networks were constructed for practical application purposes: one network without a correlation threshold resulting in a higher number of edges; and another network setting correlation thresholds at −0.5 and 0.5, leading to fewer but more relevant edges. This approach allows for comparison of networks with varying edge densities and relevance in the context of disease‐specific conditions.

#### 2.6.2 Model Construction

The drug‐disease interaction prediction model, when applied to the TCGA disease‐specific network, closely mirrors the methodology used with the Human Signaling Network. The primary distinction between these models lies in the networks utilized for conducting random walks. All other parameters, including the model architecture, training process, and evaluation criteria, remain consistent across both applications. This uniformity ensures a direct and fair comparison of the models' performance, highlighting the impact of using disease‐specific networks as apposed a more generalized human signaling network.

### 2.7 Cell Lines, Drugs, and Cytotoxicity Assay

The cell lines used in this study were purchased from Shanghai Institute of Biochemistry and Cell Biology. HGC‐27 and HCT‐15, were maintained in RPMI1640 (Gibco, Carlsbad, USA) with 10% fetal bovine serum (FBS, Gibco, Carlsbad, USA) and 1% penicillin/streptomycin (P/S) (BasalMedia, Shanghai, China). All cell lines were cultured at 37 °C in a 5% CO2 atmosphere. Cells were seeded in 96‐well plates at a density of 5000 cells per well. After 24 h, the cells were treated with increasing doses of drugs. Following a 48‐h treatment in the incubator, we determined the cell viability using a Cell Counting Kit‐8 assay. Drugs were obtained from commercial vendors and diluted to a working concentration with PBS. The half‐maximal inhibitory concentration (*IC50*) values for single drugs and synergy scores for drug combinations were calculated by SynergyFinder.^[^ ^69^ ^]^

## 3 Results

### 3.1 Method Architecture

**Figure 1:**
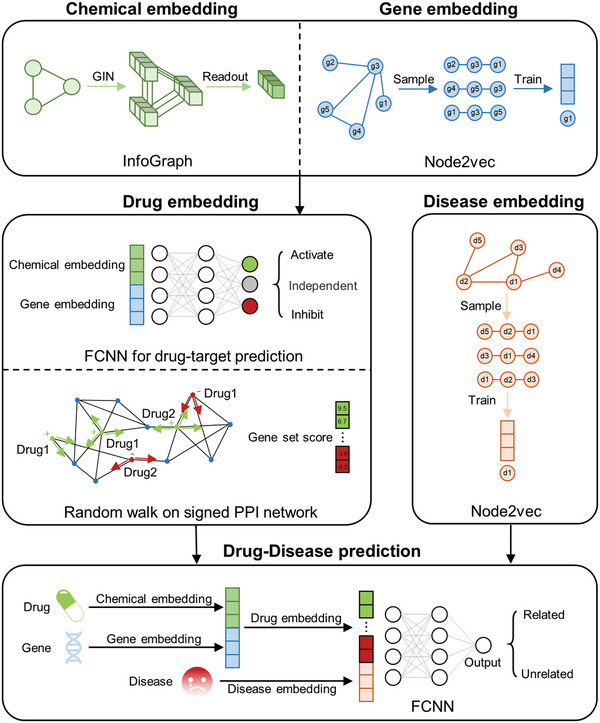
The workflow diagram depicting the methodology employed in this study. The study employs the InfoGraph method to obtain the chemical embeddings, while the node2vec method is utilized to acquire gene and disease embeddings. These embeddings are input into a fully connected neural network to predict chemical‐gene interactions. Subsequently, genes undergo random walk on the signed PPI network to obtain drug embeddings. Finally, another fully connected neural network is used to predict interactions between the drugs and diseases based on the drug and disease embeddings.

In order to construct a precise model for predicting DDIs, an initial step involved predicting the association between individual drugs and genes. This was accomplished by extracting drug features through an InfoGraph model and deriving gene embeddings from the human Protein‐Protein Interaction (PPI) network using the node2vec method. Subsequently, the concatenated drug features and gene embeddings were input into a fully connected neural network to carry out classification tasks. The findings indicate the presence of either an activating or inhibiting association between a drug and a gene. Following the acquisition of a drug's targets, they are randomly walked on the signed PPI network to gather comprehensive information on the drug's effects on the entire gene set. Disease features are represented by constructing a disease entry network based on the MeSH structure tree, and applying the node2vec algorithm. Finally, the drug action features and disease features are integrated and fed into a fully connected neural network to predict the presence of a potential relationship between them. The workflow is briefly shown in **Figure** **1**.

Considering the significant imbalance between positive and negative samples, we employed a combination of random down‐sampling of negative samples and up‐sampling of positive samples using the Synthetic Minority Over‐sampling Technique (SMOTE). The model with the highest F1 score was identified as the optimal choice during the training phase. Subsequent testing revealed the model's adaptability to drug combination tasks, as well as the potential for enhancing accuracy by customizing the random walk PPI network into disease‐specific networks.

### 3.2 Prediction for Single Drug

In this section, the model is applied to predict the correlation between individual drugs and diseases, while also comparing it with previous methods and baseline models.

#### 3.2.1 Experimental Results

**Figure 2:**
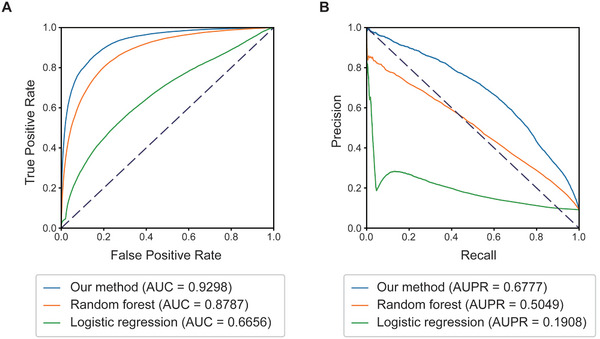
The model's performance on the prediction of single drug‐disease interactions. A) Receiver Operating Characteristic curve (ROC) of the model. B) Precision‐Recall Curve (PRC) of the model.

The predictive model for single DDI obtained an area under curve (AUC) of 0.93 (**Figure** **2**A). Despite the utilization of the SMOTE method to augment positive samples, the significant imbalance in sample types within the original dataset hindered the model's precision and recall on the validation dataset from reaching optimal levels (Figure 2B). The predictive model for single DDI obtained an AUC of 0.93 (Figure 2A). Despite the utilization of the SMOTE method to augment positive samples, the significant imbalance in sample types within the original dataset hindered the model's precision and recall on the validation dataset from reaching optimal levels (Figure 2B). However, the SMOTE method demonstrated its effectiveness in enhancing the model's performance. We compared different sampling strategies, starting with random downsampling, where we created a balanced dataset with an equal number of positive and negative samples (1:1 ratio) for both training and validation sets, and designated this approach and its corresponding model as “1x”. In contrast, the previously mentioned 10 times upsampling approach and its model were labeled as “10x”. Cross‐validation results of both datasets and models (**Table** **3**) revealed that models trained on 1x negative samples showed comparable performance to those trained on 10x negative samples when evaluated on a 1x dataset. However, on a 10x dataset, the 10x model exhibited superior performance, particularly with regard to the F1 score. This indicates that the 10x model is more effective at identifying true positives without overclassifying samples as positive, which is a common issue with the 1x model, leading to higher recall but lower precision.

**Table 3:** Cross validation between Random Sampling and SMOTE on 1x and 10x dataset.

Dataset	Model	AUC	AUPR	ACC	F1	Precision	Recall	Specificity
**1x**	**1x**	0.9259	0.9254	**0.8510**	**0.8491**	0.8608	**0.8377**	0.8645
	**10x**	**0.9286**	**0.9309**	0.8445	0.8312	**0.9091**	0.7656	**0.9234**
**10x**	**1x**	0.9270	0.6478	0.8558	0.5167	0.3712	**0.8500**	0.8563
	**10x**	**0.9298**	**0.6777**	**0.9276**	**0.6316**	**0.5867**	0.6839	**0.9519**

To further optimize our approach, we conducted a comprehensive comparison of sampling methods using the 10x dataset. We evaluated various upsampling and downsampling techniques, including random sampling, NearMiss,^[^ ^70^ ^]^ ADASYN,^[^ ^71^ ^]^ BorderlineSMOTE,^[^ ^72^ ^]^ and SMOTE (**Table** **4**). Our results indicated that upsampling methods generally outperformed downsampling methods. Among the upsampling methods, performance remained remarkably consistent, with minimal variation between different techniques. We ultimately selected SMOTE due to its optimal balance of performance, computational efficiency, and widespread adoption in addressing class imbalance issues.

**Table 4:** Comparison between different sampling method.

Model	AUC	AUPR	ACC	F1	Precision	Recall	Specificity
Random (downsampling)	0.9270	0.6478	0.8558	0.5167	0.3712	0.8500	0.8563
NearMiss (downsampling)	0.7073	0.1965	0.7390	0.2771	0.1851	0.5516	0.7577
ADASYN (upsampling)	**0.9304**	0.6742	0.9272	**0.6325**	0.5832	**0.6909**	0.9507
BorderlineSMOTE (upsampling)	0.9301	0.6706	0.9273	0.6304	0.5852	0.6832	0.9517
SMOTE (upsampling)	0.9298	**0.6777**	**0.9276**	0.6316	**0.5867**	0.6839	**0.9519**

Our approach demonstrated superior performance compared to alternative methods, particularly LAGCN and SCMFDD, which utilize similar datasets. LAGCN acquires drug and disease embeddings through the integration of drug‐disease associations, drug‐drug similarities, and disease–disease similarities within a heterogeneous network, subsequently employing a layer attention graph convolutional network for evaluation. SCMFDD projects drug‐disease associations into low‐rank spaces, incorporating drug feature‐based similarities and disease semantic similarity as constraints. Our approach demonstrates superior performance across various predictive metrics (**Table** **5**). While our study's dataset differed in having a tenfold lower proportion of negative samples compared to LAGCN and SCMFDD, the similarity in specificity between the three methods enables recall to serve as a reliable performance indicator. In scenarios where the number of positive samples remains constant while the number of negative samples vary, the recall rate remains consistent. Given comparable specificity levels, the emphasis shifts toward the model's capability to accurately detect true DDIs, where our method's superior recall rate affirms its enhanced predictive capability.

**Table 5:** Performance comparison of different methods on the drug‐disease association prediction task.

	AUC	AUPR	ACC	F1	Precision	Recall	Specificity
LAGCN	0.8750	0.3168	0.9605	0.3150	0.2800	0.3600	0.9760
SCMFDD	0.8737	0.2644	**0.9632**	0.3130	0.2954	0.3329	**0.9795**
CTST	**0.950**	0.271	–	–	–	–	–
GFPred	0.945	0.243	–	–	–	–	–
CBPred	0.926	0.163	–	–	–	–	–
LRSSL	0.831	0.107	–	–	–	–	–
MBiRW	0.828	0.045	–	–	–	–	–
HGBI	0.702	0.012	–	–	–	–	–
Ours	**0.9298**	**0.6777**	0.9276	**0.6316**	**0.5867**	**0.6839**	0.9519

(‐) indicates metrics not accessible.

Further comparation with additional methods, including CTST, GFPred, CBPred, LRSSL, MBiRW, and HGBI, demonstrated comparable AUC but superior AUPR performance (**Table** **6**), indicating better overall predictive power. Moreover, when compared to other baseline methods utilizing the same dataset and data balancing techniques, our approach showed superior performance across most metrics (**Table** **7**).

**Table 6:** Comparisons between baseline methods and our method.

	AUC	AUPR	ACC	F1	Precision	Recall	Specificity
Random Forest	0.8787	0.5049	0.9223	0.4050	**0.6633**	0.2915	**0.9852**
Logistic Regression	0.6656	0.1908	0.5887	0.2250	0.1357	0.6580	0.5818
Ours	**0.9298**	**0.6777**	**0.9276**	**0.6316**	0.5867	**0.6839**	0.9519

**Table 7:** Comparisons between zero‐shot and few‐shot models.

	AUC	AUPR	ACC	F1	Precision	Recall	Specificity
Zero‐shot model	0.7445	0.7287	0.7105	0.7412	0.6702	0.8289	0.5921
Random Forest	0.7614	0.8102	0.6447	0.6625	0.6310	0.3974	0.5921
Logistic Regression	0.7829	0.7324	0.7039	0.7368	0.6632	0.8289	0.5789
Few‐shot model	**0.8045**	**0.8239**	**0.7434**	**0.7746**	**0.6907**	**0.8816**	**0.6053**

#### 3.2.2 Ablation Study

**Figure 3:**
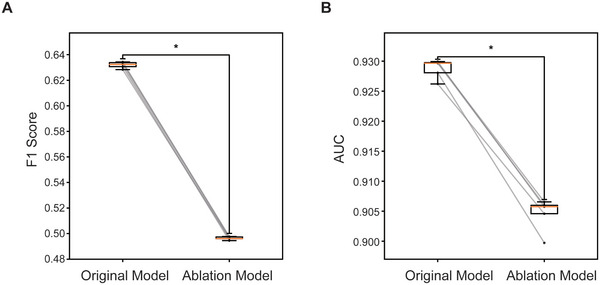
Comparison of F1 scores A), and AUC B) between the original model and the model without the drug embedding module across fivefold cross‐validation, respectively.

In our approach, two modules are employed for the extraction of drug features: 1) the chemical embedding module utilizing Infograph; 2) the drug embedding module incorporating drug‐target prediction and random walk techniques. While the chemical embedding module is proficient in extracting drug features for the final prediction, the inclusion of the drug embedding module is deemed necessary to enhance the extraction efficacy. An ablation study was conducted to assess the impact of the drug embedding module. In this study, fivefold cross‐validation was employed to assess the performance of two models‐one with a drug embedding module and one without‐using the same dataset. The SMOTE method was consistently applied to augment the training set in each experiment. The results demonstrated that the original model exhibited superior performance in terms of F1 score and AUC compared to the model lacking the drug embedding module (**Figure** **3**). This suggests that the incorporation of the PPI network in the drug embedding module enables the extraction of more comprehensive and significant drug features.

### 3.3 Prediction for Drug Combinations

In this section, we apply the model to predict the correlation between combination drugs and diseases and investigate the factors contributing to the enhanced predictive accuracy in comparison to individual drugs.

#### 3.3.1 Experimental Results

The model, initially trained using single‐drug data, was subsequently utilized for the analysis of drug combinations. In order to enable a comprehensive comparison of efficacy differences between single drugs and drug combinations, a total of 104 therapies were chosen, ensuring that each drug‐disease pairing was also present in the drug‐disease interaction dataset. Predictions were generated for each combination drug therapy under two conditions: 1) direct prediction of the drug combination, and 2) individual prediction of each drug within the combination, with the resulting averages being calculated.

**Figure 4:**
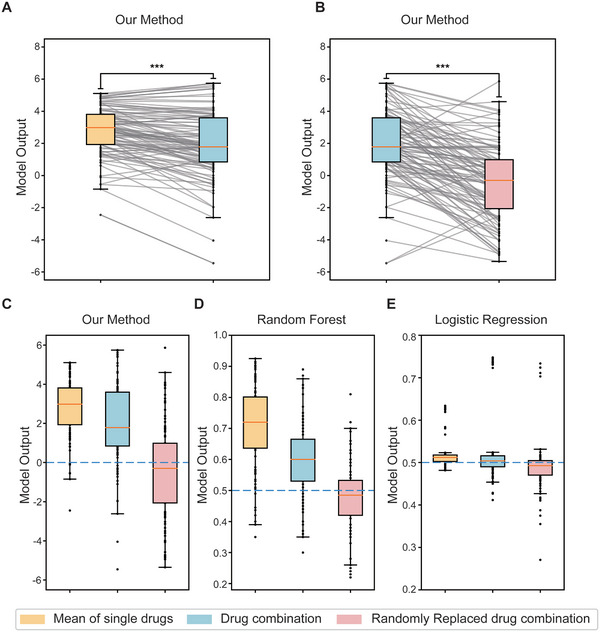
The performance of the model in predicting interactions between drug combinations and diseases. A) For each drug combination, the boxplot shows the comparison between the average outputs of individual drugs and the combined output of the drug combination. B) For each drug combination, the boxplot compares the direct output of the drug combination with the output of the adjusted drug combination, where a random drug is substituted randomly. C–E) The performance of the efficacy of various methods on three distinct metrics: 1) the average outputs of individual drugs in a drug combination, 2) the direct output of the drug combination as a whole, and 3) the output of modified drug combination in which a randomly selected drug is replaced.

The results showed that among 104 combination drug therapies, 76 predictions yielded lower values compared to the average of single drug predictions (*P*‐value = 1.07 × 10^−9^), suggesting that the current model exhibits enhanced predictive capabilities for single drugs (**Figure** **4**A). In order to assess the generalizability of the model to combination drugs, a random drug within the drug combination was replaced with a randomly selected drug from a pool of 7940 drugs. The comparison of predictions before and after the replacement revealed that 95 out of 104 combination drug therapies exhibited superior outcomes prior to the replacement. Additionally, a significant drop in predictions was observed for the majority of drug combinations following the replacement (*P*‐value = 1.23 × 10^−14^), thereby validating the predictive capabilities of the model for drug combinations (Figure 4B).

Furthermore, in order to assess the effectiveness and accuracy of various single drug‐disease interaction prediction methods when applied to the prediction of drug combinations, this study also examined the performance of random forest and logistic regression models that were trained on the single drug dataset. The results demonstrate that the random forest method shows inferior performance in predicting drug combinations, while the results of the logistic regression method are closely aligned in three scenarios, posing challenges in distinguishing them (Figure 4C–E). Overall, our approach demonstrates superior predictive capabilities for both single drug and drug combination outcomes.

#### 3.3.2 Few‐Shot Study

In this section, we leverage the capabilities of few‐shot learning to enhance the model's effectiveness in discerning interactions between various combinations of drugs and diseases. Initially designed for predicting outcomes in individual drug‐disease pairings, the model undergoes a sophisticated fine‐tuning procedure utilizing a dataset containing 301 drug combination therapies. This dataset is meticulously divided into a training subset comprising 225 instances and a validation subset consisting 76 instances. To address the challenge posed by the exclusively positive nature of the samples, we devised a method to create corresponding negative samples for each subset. This involves the deliberate modification of one drug within each combination in a random manner.

**Figure 5:**
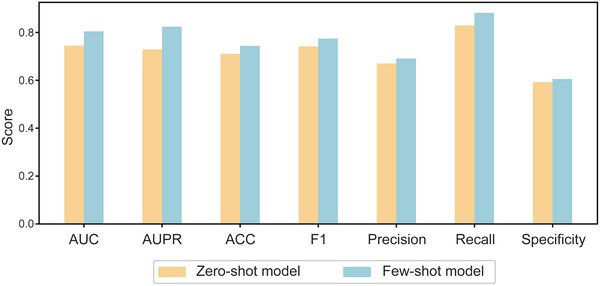
A comparison between the zero‐shot and few‐shot models across various metrics within the context of drug combination datasets.

Despite the fact that the dataset utilized for fine‐tuning comprises only 0.032% of the initial training dataset, the model trained using few‐shot learning techniques shows a significant improvement in performance (**Figure** **5**). The enhanced performance is evident across a broad spectrum of metrics, markedly outperforming the baseline established by the zero‐shot model (Table 7). The findings clearly demonstrate that through fine‐tuning, the model achieves a notably higher level of precision in predicting the intricate relationships between drug combinations and diseases. This underscores the significant impact of few‐shot learning on enhancing the model's predictive capabilities, even when confronted with limited supplementary training data.

#### 3.3.3 Case Study

In this section, we conducted cell biology experiments to validate our prediction of the relationships between drugs or drug combinations and diseases. We carefully selected a group of chemicals with potential therapeutic effects against cancer and conducted thorough experiments on cell lines to confirm their effectiveness. Initially, we utilized a database to identify drugs that have demonstrated efficacy in cancer treatment, pairing them with specific types of cancer to establish a set of validated drug‐cancer relationships. Subsequently, each drug in the drug dataset was systematically matched with every type of cancer in the disease dataset to create a comprehensive set of hypothetical pairs. The single drug‐disease interaction model was then utilized to assess these pairings, identifying the top 10% as potential drug‐cancer interactions. These potential interactions were integrated with the confirmed interactions to produce a comprehensive list of potential drug‐cancer interactions. To further narrow down the selection, one drug from the confirmed pairs and another from the potential pairs were chosen to create drug combinations. These combinations were subsequently evaluated using a refined model to determine the most effective drug combinations.

From the pool of highly effective drug combinations, we specifically selected two prevalent cancer cell lines as the subjects of our experimental inquiries. Each selected cell line intentionally matched with a clinically approved drug and an unapproved drug, with the objective of investigating innovative therapeutic approaches rooted in established pharmacological principles (**Table** **8**). This methodical selection procedure ultimately led to the discovery of two drug combinations for in‐depth experimental evaluation.

**Table 8:** Cell lines chosen for drug combination assays.

Disease	Cell line	Approved Drug	Novel Drug
Colonic Neoplasms	HCT‐15	Oxaliplatin	Cytarabine
Stomach Neoplasms	HGC‐27	Docetaxel	Cytarabine

**Figure 6:**
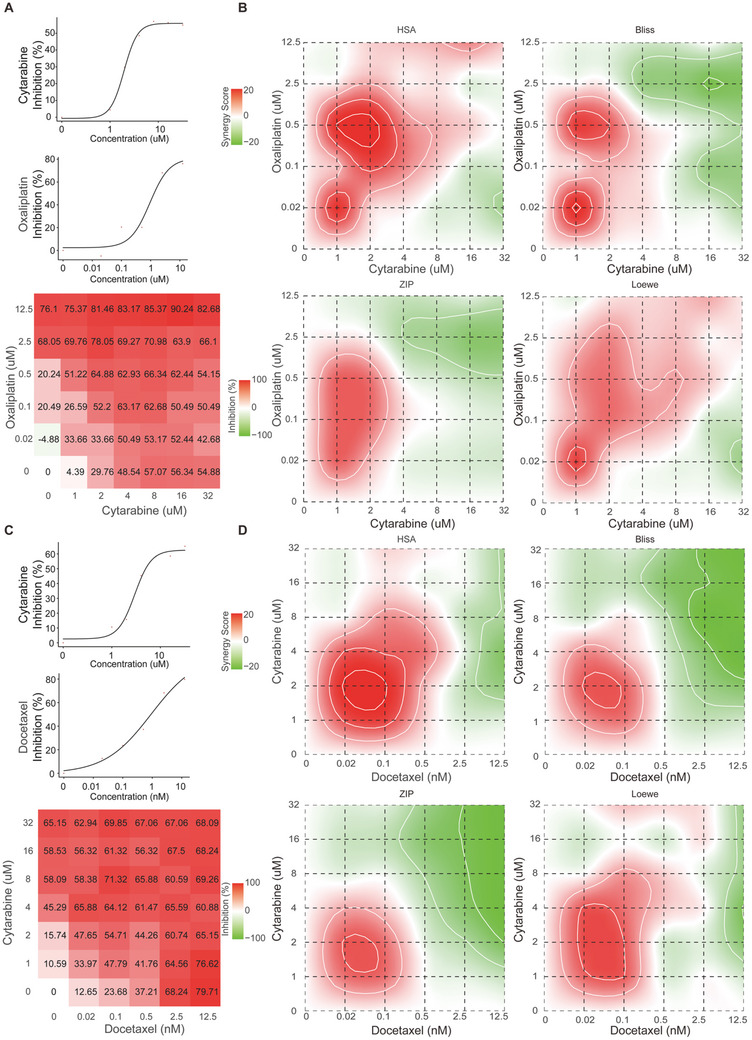
Cell growth inhibition by specific drugs, and the synergistic inhibitory effects observed in drug combinations. A) The cytotoxic effects of cytarabine, oxaliplatin, and their combination were analyzed in HCT‐15 cells. B) Heatmaps displaying synergy scores of the response to a multi‐dose drug combination in HCT‐15 cells. C) Cytotoxic analysis of cytarabine, docetaxel, and their combination was conducted in HGC‐27 cells. D) Heatmaps showing synergy scores of the response to a multi‐dose drug combination in HGC‐27 cells. A synergy score exceeding 10 suggests potential synergistic effects between the two drugs.

Our study revealed that cytarabine demonstrates therapeutic potential for colonic neoplasms and gastric cancer. Furthermore, we observed a synergistic effect when combining cytarabine with docetaxel and oxaliplatin, both first‐line drugs for gastric cancer and colon cancer, respectively (**Figure** **6**). These predictive results have been validated through experimental investigation.

To validate our approach, we expanded our analysis of biological significance through literature evidence and computational validation, identifying several key synergistic interactions between antineoplastic and non‐antineoplastic drugs. The Mitomycin‐curcumin combination inhibits MCF‐7 cell proliferation and cell cycle progression via the p38 MAPK pathway, as previously reported.^[^ ^73^ ^]^ Our computational analysis, which used enrichment analysis of differentially expressed genes identified through PPI network propagation, confirmed this finding, showing enrichment in the Ras signaling pathway (*P*‐value = 1.43 × 10^−17^) and cellular secretion processes. GO term analysis revealed modulation of chemical synaptic transmission (*P*‐value = 1.71 × 10^−10^) and positive regulation of secretion (*P*‐value = 7.51 × 10^−11^), indicating multiple coordinated mechanisms(Tables S1 and S2, Supporting Information). For Cisplatin and lupeol, known to synergistically inhibit hepatocellular carcinoma through the PI3K‐Akt pathway,^[^ ^74^ ^]^ our analysis showed strong enrichment in this pathway (*P*‐value = 7.17 × 10^−78^). We also found enrichment in MAPK cascade regulation (*P*‐value = 9.97 × 10^−56^) and cytosolic calcium ion concentration regulation (*P*‐value = 2.11 × 10^−44^), suggesting broader cellular response networks(Tables S3 and S4, Supporting Information). The Sorafenib and Sulfasalazine combination analysis revealed novel insights, with significant enrichment in cancer‐related pathways (*P*‐value = 6.66 × 10^−21^) and proteoglycans in cancer (*P*‐value = 1.08 × 10^−16^). GO term analysis highlighted activation of the AMPK signaling pathway (*P*‐value = 2.81 × 10^−12^) and cell‐cell adhesion processes, indicating potential new synergistic mechanisms (Tables S5 and S6, Supporting Information).^[^ ^75^ ^]^

### 3.4 Prediction on Disease‐Specific Networks

In this section, we replaced the random walk network of the model with the computed cancer‐specific network and assessed its efficacy in enhancing drug prediction accuracy for particular diseases. A disease‐specific network was constructed for each TCGA cancer, and drugs for treating the cancer were selected. These drugs were paired with the cancer to create positive samples, while an equivalent number of other drugs were randomly chosen and paired with the cancer to create negative samples. The samples were divided into a training set comprising 80% of the data and a validation set comprising 20% of the data. The prediction models were trained using three PPI networks for each type of cancer. The results showed that, among the 18 cancers studied, 13 exhibited improved performance when utilizing disease‐specific networks, while the remaining 5 demonstrated comparable results (**Table** **9**). These results suggest that disease‐specific networks hold promise for enhancing the accuracy of DDIs predictions.

**Table 9:** The model's performances under the common network and disease‐specific networks.

Cancer Type	Common network	Cancer specific network (threshold = 0.5)	Cancer specific network (threshold = 0)
Sarcoma	63.16%	**73.68%**	**73.68%**
Stomach Adenocarcinoma	63.64%	65.45%	**69.09%**
Bladder Urothelial Carcinoma	62.50%	61.11%	**63.89%**
Testicular Germ Cell Tumor	64.29%	**78.57%**	71.43%
Ovarian Serous Cystadenocarcinoma	50.00%	50.00%	**75.00%**
Mesothelioma	73.68%	**78.95%**	**78.95%**
Esophageal Carcinoma	65.52%	**82.76%**	68.97%
Head & Neck Squamous Cell Carcinoma	45.45%	45.45%	**54.55%**
Pheochromocytoma & Paraganglioma	33.33%	**44.44%**	33.33%
Breast Invasive Carcinoma	71.43%	71.43%	**85.71%**
Liver Hepatocellular Carcinoma	64.95%	62.89%	**69.07%**
Colon Adenocarcinoma	52.86%	**57.14%**	51.43%
Lung Adenocarcinoma	67.67%	**72.18%**	68.42%

## 4 Discussion

To address the challenges in drug development and improve therapeutic outcomes, we propose a biologically network‐based model that predicts interactions among drugs, diseases, and combination therapies. The main goal of this study is to optimize the drug development process by identifying novel indications for current medications and more effective treatment combinations for various diseases.

Our innovative approach adeptly tackles the challenge of balancing large‐scale positive and negative samples, resulting in enhanced performance across diverse evaluation metrics including AUC, area under the precision‐recall curve (AUPR), and F1 score compared to prior methodologies. Unlike conventional methods that primarily rely on drug and disease features or deep learning methodologies, our approach extends beyond these strategies by incorporating network techniques that utilize massive existing knowledge. This approach enables the derivation of more precise and informative drug features. By utilizing network target theory, we conducted simulations to analyze the impact of drugs on biological networks, with a particular focus on drug network targets as crucial components, resulting in enhanced outcomes. A notable strength of our model lies in its capacity to forecast not only individual drug‐disease interactions but also interactions between combined therapies and diseases. This functionality presents novel opportunities for personalized medicine by enabling treatment plans tailored to individual disease profiles. As molecules exhibit tissue‐specific expression patterns, we evaluated our model within disease‐specific biological network contexts, establishing it as an innovative tool for future investigations. Through the substitution of random walk networks, our approach facilitates predictive analysis across various disease scenarios while maintaining high levels of accuracy and efficiency. The integration of network methodologies in our model harnesses well‐established biological insights pertaining to molecular pathways, protein‐protein interactions, gene expression profiles, and other relevant data sources. Furthermore, the analysis of complex interconnections within the biological system can improve the predictive accuracy of our model and offer valuable insights into the mechanisms driving drug effectiveness. Our analysis of the cytarabine‐docetaxel combination revealed significant enrichment in the mTOR signaling pathway (*P*‐value = 6.64 × 10^−9^) and Ras signaling pathway (*P*‐value = 6.89 × 10^−9^), suggesting synergy through the simultaneous targeting of key cancer pathways involved in tumor growth and survival in gastric cancer. Additionally, enrichment in G protein‐coupled receptor binding (*P*‐value = 2.92 × 10^−7^) indicates the potential for modulation of receptor‐mediated signaling, which may further enhance the therapeutic effects (Tables S7 and S8, Supporting Information). In our analysis of cytarabine‐oxaliplatin, we observed significant enrichment in the cAMP signaling pathway (*P*‐value = 5.20 × 10^−48^) and cGMP‐PKG signaling pathway (*P*‐value = 6.94 × 10^−24)^, indicating synergy through the modulation of essential cellular processes such as proliferation and apoptosis in colorectal cancer. Moreover, enrichment in the adenylate cyclase‐modulating G protein‐coupled receptor signaling pathway (*P*‐value = 6.42 × 10^−50^) suggests additional effects on tumor growth and metastasis, supporting the combination's broader impact on disease progression (Tables S9 and S10, Supporting Information).

Despite its demonstrated effectiveness, our study has several limitations that warrant discussion. First, the multi‐stages methodology may introduce data loss and error accumulation, potentially affecting final indicators. To address this issue, future work should incorporate additional dimensions during initial of drug and disease feature extraction to preserve more detailed information and structural integrity. Second, our current approach relies on a simple fully connected neural network for drug target prediction. While effective, integration of more cutting‐edge prediction algorithms may potentially improve accuracy.

Our random walk‐based approach, though successful in simulating drug therapy effects, could benefit from more sophisticated computational frameworks. Recent advances in dynamic multi‐objective optimization algorithms offer promising directions for enhancement. For instance, Li et al. proposed a hierarchical response system that could be valuable for modeling varying intensities of drug‐target interactions.^[^ ^76^ ^]^ Similarly, Li et al. developed a population robustness‐based switching response framework particularly relevant for simulating combination drug therapies where multiple drug effects must be considered simultaneously.^[^ ^77^ ^]^ While our current random walk implementation provides a solid foundation, incorporating principles from these advanced optimization strategies could enhance our ability to model complex therapeutic scenarios. An immediate improvement could involve increasing both the quantity and duration of walks to reduce model randomness and generate more stable, precise drug action features.

Although our model demonstrates strong predictive power for drug combination efficacy, several limitations present opportunities for future development. A significant limitation is that our model does not directly address drug‐related side effects, which are crucial for clinical applications. To enhance clinical relevance, we plan to extend our framework in several ways. First, we will integrate comprehensive side effect databases (SIDER and FAERS) to incorporate known adverse effects into our prediction framework. Second, we aim to develop sophisticated scoring metrics that simultaneously evaluate therapeutic efficacy and potential side effects, enabling more balanced assessments. Third, we will expand pathway analysis to include molecular mechanisms associated with common drug side effects, providing mechanistic insights into potential adverse reactions. These enhancements will require experimental validation to ensure reliability.

Another important direction for future research is the optimization of dosage in drug combinations. While our current results suggest that synergistic combinations may allow for reduced drug dosages while maintaining therapeutic efficacy, systematic studies are needed to quantify these effects and establish optimal dosing regimens. This will require both computational modeling of dose‐response relationships and experimental validation in relevant model systems. These planned improvements will help bridge the gap between computational predictions and clinical applications, ultimately contributing to more effective and safer combination therapies.

In summary, our model accurately predicts interactions between individual drugs and their combinations with diseases, particularly in various types of cancer; demonstrating exceptional capability in managing extensive datasets and identifying relevant drug features. We propose that this approach holds significant potential for enhancing drug development by improving the accuracy and effectiveness of predictions regarding associations between drugs and diseases.

## Conflict of Interest

The authors declare no conflict of interest.

## Author Contributions

Q.L., Z.C., B.W., and B.P. contributed equally to this work. L.L. and S.L. conceived and supervised the study and finalized the manuscript. Z.C., Q.L., B.W., W.Z., and T.Z. performed data processing, model design, and computational analysis. Z.C. participated in the study design and drafted the manuscript. B.P. and M.S. conducted the in vitro experiments. Z.Z. engaged in the model debugging. All authors read and approved the final manuscript.

## Supporting information



Supporting Tables

## Data Availability

The data that support the findings of this study are available from the corresponding author upon reasonable request.
